# Housing inequality and settlement persistence are associated across the archaeological record

**DOI:** 10.1073/pnas.2400696122

**Published:** 2025-04-14

**Authors:** Dan Lawrence, Amy Bogaard, Gabriela Cervantes Quequezana, Francesca Chelazzi, Gary M. Feinman, Adam S. Green, Helena Hamerow, Jessica Munson, Scott G. Ortman, Amy E. Thompson

**Affiliations:** ^a^Department of Archaeology, Durham University, Durham DH1 4HW, United Kingdom; ^b^School of Archaeology, University of Oxford, Oxford OX1 3TG, United Kingdom; ^c^Santa Fe Institute, Santa Fe, NM 87501; ^d^Department of Anthropology, University of Pittsburgh, Pittsburgh, PA 15260; ^e^Department of Asian and North African Studies, Ca’Foscari University, Venice 30123, Italy; ^f^Neguanee Integrative Research Center, Field Museum of Natural History, Chicago, IL 60605; ^g^Department of Anthropology, University of Illinois-Chicago, Chicago, IL 60607; ^h^Department of Archaeology, University of York, King’s Manor, York YO1 7EP, United Kingdom; ^i^Department of Environment and Geography, University of York, King’s Manor, York YO1 7EP, United Kingdom; ^j^Department of Anthropology-Sociology, Lycoming College, Williamsport, PA 17701; ^k^Institute of Behavioral Science, University of Colorado, Boulder, CO 80309; ^l^Department of Geography and the Environment, The University of Texas at Austin, Austin, TX 78712

**Keywords:** sustainability, inequality, persistence, archaeology

## Abstract

Definitions of sustainability commonly stress both systemic continuity and equality over time. However, the degree to which these two sides of sustainability might be related has not been systematically investigated. Recent theoretical and methodological insights have provided archaeologists with new tools for investigating sustainability in premodern societies. Here, we use Gini coefficients on residence size measurements as an estimate of material inequality and information on the persistence of settlements as a measure of continuity. Persistence is derived from the length of time a settlement endured in a particular state, and we assume that higher levels of persistence indicate greater adaptive capacity and resilience. We articulate an approach which allows us to combine these proxies to investigate the relationship between these two sides of sustainability and apply this to a global dataset of settlements from the last 10,000 y. We find that persistence is weakly correlated with material inequality, rather than equality, overall. We also find that this relationship grows stronger as settlement systems increase in scale and is strongest for larger sites in larger systems. This pattern may be the result of both increased functional diversity at a site level and network effects whereby residents of larger settlements were able to claim a larger share of the surplus produced by the settlement system.

## Significance

Ensuring the sustainability of human systems is a major challenge facing humanity. Preindustrial societies can provide a rich dataset to assess the drivers of sustainability over time. We investigate the relationship between the duration of occupation (persistence) and the degree of material inequality from a global dataset of past settlements. Our results demonstrate that higher material inequality is correlated with higher persistence, but they are not causally associated. Instead, both rise with the increased scale and complexity of human systems. Our results suggest interventions to reduce material inequality can be accomplished without reducing persistence but should attend to systemic effects of scale.

The United Nations Bruntland Commission (1) is credited with establishing the idea of sustainable development in policy and popular discourse (2). The commission defined sustainability as “meeting the needs of the present without compromising the ability of future generations to meet their own needs.” This definition incorporates two separate ideas, systemic continuity and social equality, which are now recognized as fundamental to the future of humanity in the Anthropocene (3, 4), even by critics of the original report (5). However, the relationship between these two sides of sustainability has not been systematically investigated. This is in part because such an analysis would require long-run information on the continuity of particular social formations. The historical sciences are in a unique position to identify such patterns, providing us with a rich archive of successes and failures at a range of scales, from individual households to settlements, cities, states, and social systems, which should be highly informative. Research from the historical sciences has had a limited impact on frameworks such as the Assessment Reports of the Intergovernmental Panel on Climate Change or United Nations Sustainable Development Goals Reports. Where they are included, archaeological contributions have focused on the protection and management of heritage assets in the present, rather than as a source of knowledge on socioecological interactions in the past. This absence of information on the human past from modern research and policy debates is of increasing concern, within both the historical sciences themselves (6–8) and the sustainability community (9, 10).

Recent theoretical and methodological insights have provided archaeologists with new proxies for investigating these two sides of sustainability in preindustrial societies: the persistence of settlements over time as a measure of continuity, and statistical and analytical methods for understanding past wealth distributions based on disparities in residential unit size. Here, we use these two proxies to investigate two interrelated questions. First, are there systematic relationships between the duration of occupation and level of material inequality in preindustrial settlements, and second, what factors might account for these relationships? We use the historical record to address a third question: What can these relationships tell us about the role of material inequality in facilitating sustainability in the present?

## Settlement Persistence and Material Inequality.

The concept of settlement persistence has gained traction in archaeology, particularly among scholars interested in urbanism, as a way of articulating analyses of past societies in relation to sustainability science. Scholars have also used persistence to examine urban resilience, the capacity to rebound from some exogenous shock (11, 12). The justification for settlement persistence as a proxy for sustainability is built on the insight that “a sustainable system is one which survives or persists … Sustainability, at its base, always concerns temporality, and in particular, longevity” (13). As such, the duration of an entity in a particular state, for example a settlement being occupied, can be taken as a measure of the entity’s sustainability. By comparing the relative duration of settlements against other variables, we can identify associations which might give us insight into drivers for sustainability. For example, greater longevity of cities in pre-Hispanic Mesoamerica has been demonstrated to correlate with more collective forms of governance and urban infrastructure (14, 15), while in Southwest Asia, longevity varies across different land use zones, with shorter durations in regions which require significant labor and landesque capital investment to maintain production (16). Global comparisons have shown significant variability in median durations for settlements in different regions of the world, perhaps associated with environmental attributes and productivity (17).

Persistence provides a route into one aspect of sustainability, continuity but cannot provide insights into the other half of the Bruntland definition, equality. This second side of sustainability has been neglected in archaeological work on this topic. However, understanding for whom a society was sustainable is critical if we want to draw appropriate and ethical lessons from the past for the future. For example, a long-lived city or society which relied on enslaved labor may not be one which we should seek to emulate. The study of inequality and hierarchy in the past predates the discipline of archaeology (18) and has been a major research question ever since, but here too recent work has provided new theories and proxies which mean an analysis at scale is timely. Traditional frameworks for understanding hierarchy relied on taxonomic approaches, in which societies were classified into evolutionary stages. This has long been recognized as problematic but has proved very difficult to leave behind completely, in part due to the absence of alternative theoretical models from which to make sense of archaeological data (19). Recent approaches have shown that shifts in complexity are not linear or uniform, that forms of inequality and social organization can be flexible and temporary, and that traditional associations between inequality, urbanism and polity scale cannot be taken for granted (20–22). As such, we need to disentangle the core dimensions of inequality, governance, and complexity more generally (23). Here, we focus on material inequality at an individual settlement level.

In common with the other papers in this Special Feature, we use differences in the size of residences as our measure of inequality. It is important to be precise about how we think this proxy works. Inequality, in both the past and present, is a multifaceted concept which can include economic, political, social, and cultural dimensions. Bowles et al. (24) provide a useful framework which distinguishes three broad forms of wealth, material (land, livestock, and physical possessions), relational (social ties and obligations), and embodied (physical capacities and knowledge), which can be unevenly distributed in a population to produce inequalities. Archaeologists have tended to assume that residence size should be associated with the material category, such that it can be taken as a measure of household wealth (25, 26). In a paper in this Special Feature (27), Ortman et al. use comparisons with modern housing data to argue that residence size may be better considered a proxy for household income, so a socioeconomic rate rather than an accumulated stock of assets. In either case, the claims made in this paper should be taken to refer to material inequality. We compute Gini coefficients for residential units across a subset of the sites available in the GINI project database (28, 29). Our subset includes all sites where we also have robust data on settlement duration (see *SI Appendix*, Table S1 for further details on the dataset for this paper). Further work could investigate patterns in the distribution of other forms of wealth. For example, burial datasets may be a better proxy for relational wealth, since mortuary contexts are one of the few instances in which we can directly associate an individual with specific aspects of material culture, ritual practice, and past behavior. Information from skeletal remains can also be used to reconstruct health and even well-being and therefore embodied wealth (30).

## Results

**Fig. 1.**
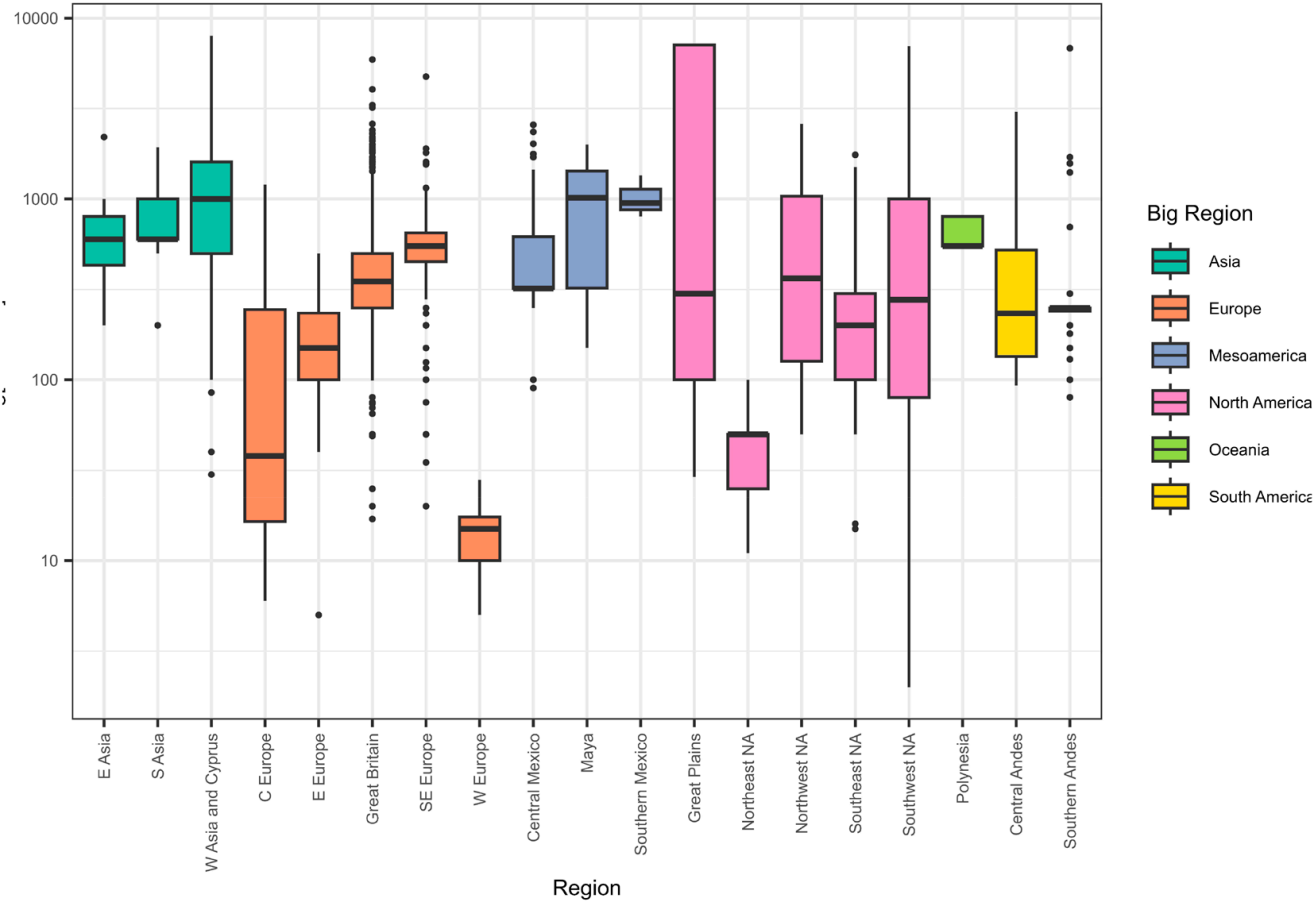
Boxplot of durations of occupation (persistence) by macroregion and region.

We first compared mean persistence (site duration) for groups of sites at two spatial scales available in the GINI database: macroregions, which correspond to continents, and regions, which represent subsets of continents with shared physical and cultural characteristics (Fig. 1 and *SI Appendix*, Table S1). Persistence varies between macroregions and regions, from a mean of 14 y in the Western Europe region to 2,177 y in the American Great Plains. This accords with published comparative analyses (17, 31), although our range is even wider than previously reported, likely due to the greater global coverage. There are several factors which determine regional persistence levels, including subsistence strategies, local environmental affordances, and resources such as building materials, forms of social organization, and cultural traditions. For example, the long-lived “tell” sites which occur across much of Southeast Europe and Southwest and Central Asia are found in lowland agricultural landscapes and have been associated with specific systems of land tenure (32), as well as an “historical ideology of dwelling” which promotes continuity (33). By contrast, the Cholistan region in the Indus River Basin demonstrates significant turnover of sites over comparatively short timescales, attributable to regular shifts in local hydrological networks (34). Our ability to recognize persistence is also affected by archaeological practice, sampling biases, and the nature of the preserved record. For example, in parts of the world with well-established tree ring chronologies, such as the Southwest United States, sites can be very precisely dated to specific years. In the Wyoming hut circles present in our Great Plains dataset, the low volume of artifacts and reliance for dating on slow-changing chipped stone assemblages (35) result in very long phases, meaning we are more likely to miss shorter periods of site abandonment. Differential persistence is a topic which merits further study, but here we are more concerned with its effects on our ability to compare between persistence and material inequality. In order to render the dataset comparable, we use the difference between individual site duration and the mean site duration of each region to compute a relative persistence score for each site. This has the effect of centering each region, such that sites with a value greater than one are more persistent than the mean and sites with a value of less than one are less persistent than the mean.

**Fig. 2.**
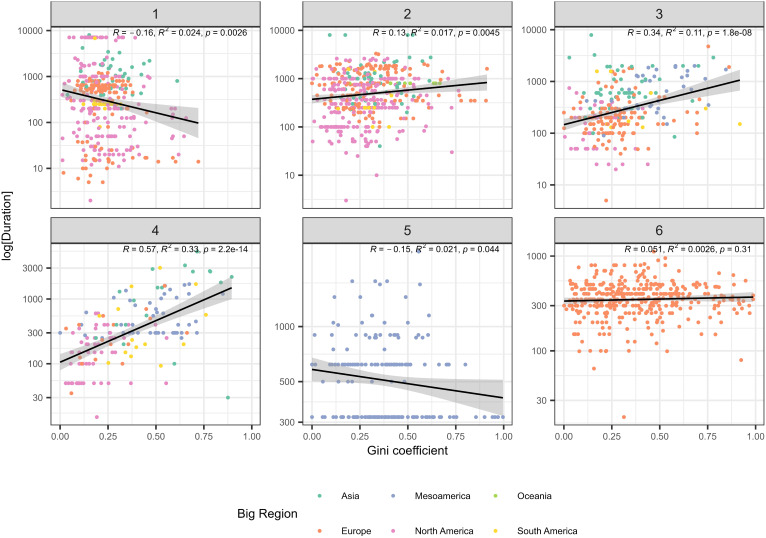
Scatter plots of duration of occupation (persistence) and Gini coefficient divided into the six [NOfLevels]. Note that levels 5 and 6 are only represented by single periods in single regions (Classic Maya for five and Roman Britain for six).

**Fig. 3.**
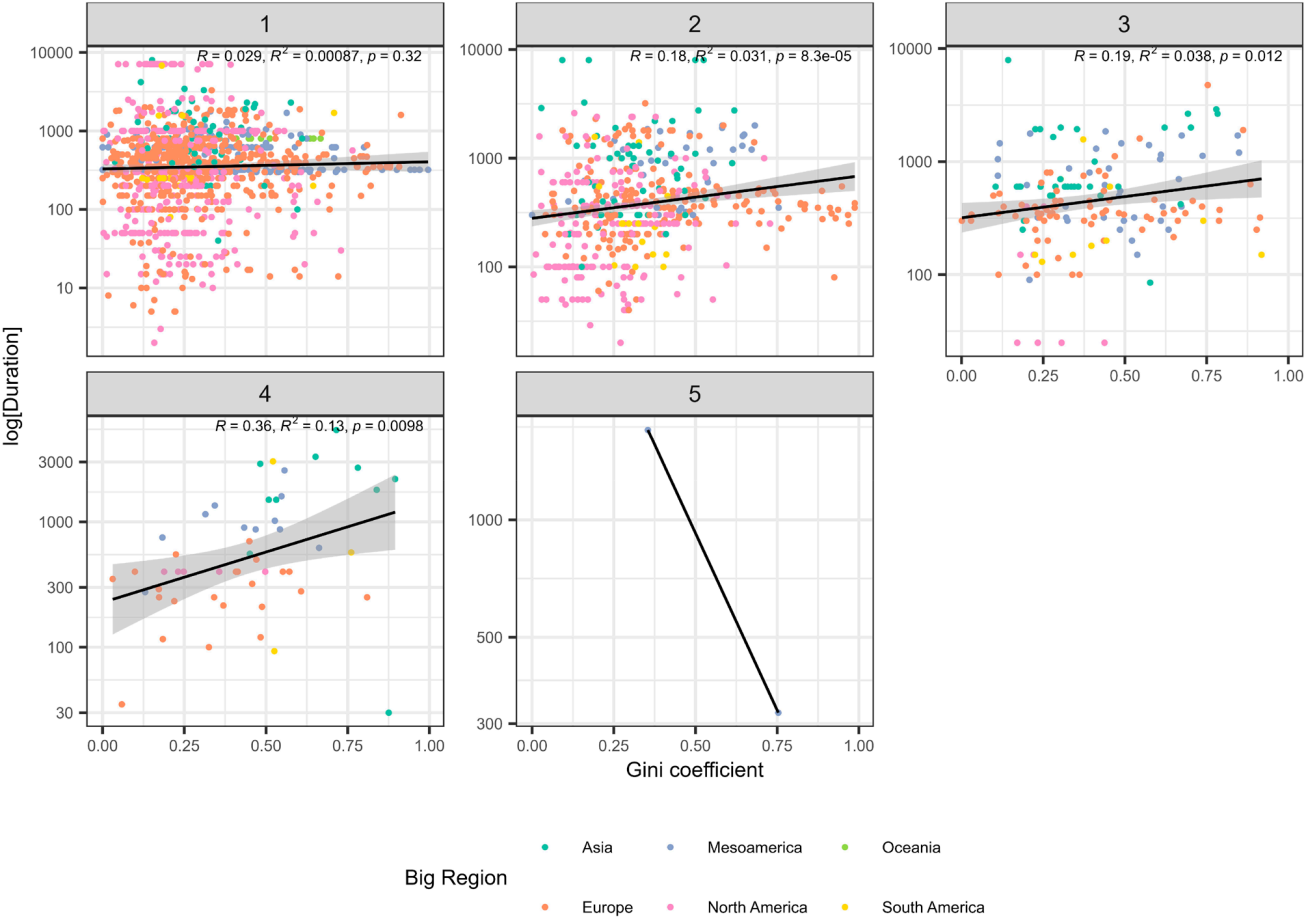
Scatter plots of duration of occupation (persistence) and Gini coefficient divided into the five [WhichLevel] scale. Note that level 5 is only represented by a single period in a single region (Classic Maya). There are no settlements in our dataset with a [Which Level] score of six. Roman Britain is the only period and region with six levels, with Rome at the top, but this site is not included in our dataset.

Overall, the Gini value and the log of relative persistence value for individual sites are weakly positively correlated, suggesting that more persistent sites exhibit greater degrees of residential disparity. We use log-transformations to show the relationships between Gini values and persistence because duration distributions are generally heavy-tailed, with a cluster of values around the mean and a large number of outliers (17). *SI Appendix*, Fig. S1 shows the relationship overall (main graph) and grouped by macroregion, with linear regression lines produced using the ordinary least squares method. Positive correlations are also visible in each microregion except Mesoamerica, where the slope is very slightly negative. Previous work on persistence has demonstrated that site size and site centrality are correlated with greater persistence (17, 36), variables which have also been demonstrated to explain residential disparities in the GINI dataset (29). For these reasons, we compared persistence across a range of variables coded in the GINI dataset, or derived from the coded variables, which relate to settlement size and organization. We first used [NOfLevels], which refers to the number of different size levels in a settlement system, and [WhichLevel], which refers to the level rank of a settlement within the system. For example, a system with villages, towns, and a city would have three levels, with villages assigned to level one, towns to level two, and cities to level three. If there were two sizes of village, large and small, then the system would move to four levels and towns and cities would move to levels three and four respectively. Fig. 2 shows the Gini value and the log of relative persistence value, divided by [NOfLevels]. Fig. 3 uses the same axes but divided by [WhichLevel]. At level one, both relationships are negative, meaning persistence decreases with increasing Gini. However, for all sites in ranked systems (where [NOfLevels] is greater than one) and all sites above the lowest level in a hierarchy (where [WhichLevel] is greater than one), persistence and material inequality are positively correlated. The strength of this relationship (represented by a higher coefficient and therefore a steeper slope) increases as you move up the levels, particularly for [NOfLevels] (see *SI Appendix*, Fig. S2 for a combined plot of the linear regression coefficients (the slope of the regression line) for Gini and persistence, which captures this trend). Because both [WhichLevel] and [NOfLevels] behave in similar ways, we also make use of a combined variable created by summing them together, called the Social Advantage score (or [SA], see ref. 25). The SA score shows a slight positive relationship with persistence in the overall trend, but there is variation in the macroregions, with North America and Europe showing significant negative relationships (*SI Appendix*, Fig. S3). Both of these regions have fewer large centralized urban centers, while in North America the subset of small sites with very long durations from the Great Plains have a large effect. In order to investigate the role of settlement size as a factor in determining persistence, we use the variable [MaxHH], or the maximum number of households estimated to have been contemporaneously occupied at each site. [MaxHH] is positively correlated with persistence in the overall trend and in every microregion (*SI Appendix*, Fig. S4).

The regression results demonstrate that measures of population, settlement hierarchy, and residential disparity affect persistence, although the fraction of the total variance in persistence accounted for by these variables is generally quite low. To test this further, we undertook linear mixed modeling using relative persistence as the dependent variable, a combination of SA score, settlement population (in households, [MaxHH]), Gini values and time in relation to the earliest agriculture ([DeltaCultivation] in the database) as fixed effects, and macroregion as a random effect (as laid out in ref. 29, this effectively means we allowed the intercepts to vary by macroregion). Our results (Table 1) show that the fixed effects can account for 51% of the variance in the dataset, and fixed and random effects combined can account for 63%. We also ran the model with the fixed variables separately for each macroregion. This demonstrated that the fixed variables are particularly effective in the Americas, explaining 91% of the variance in North America, 63% in Mesoamerica, and 50% in South America but weaker in Asia (14%), and Europe (9%). In the overall model, as expected, SA score and Gini values are positively correlated with persistence, while population [MaxHH] is slightly negatively correlated. [DeltaCultivation] has a negative slope in the overall model and in each region except Europe. This may be related to accelerating changes in material culture over time, which allows for more precise dating, shorter phase lengths and an increased ability to identify periods of abandonment. This relationship is not visible in Europe because the older sites in the dataset come from regions where very precise tree-ring chronologies are available and sites tend to be very short-lived. The linear mixed modeling results provide a robustness check on the graphed regressions. For example, the fundamental bivariate relationship between inequality and duration is shown in Table 1, Model 1, where the (significant) effects of two measures related to site size ([SA] and [MaxHH]), as well as regional differences, are held statistically constant. The partial slope for Gini here (0.07, *P* = 0.06) confirms the relationship visible in *SI Appendix*, Fig. S1.

**Table 1.** Experimental regression results, world sample, and macroregional subdivisions

Model	Spatial scope	Effect	Group	Term	Estimate	SE	Statistic	df	*P* value	R^2^m^*^	R^2^c^†^
1	World									0.512	0.626
		fixed	NA	(Intercept)	0.201	0.239	0.840	6.010	0.433		
		fixed	NA	DeltaCult_norm	−1.039	0.036	−29.191	862.597	<2e-16		
		fixed	NA	SA_norm	0.275	0.063	4.346	856.971	1.49E-05		
		fixed	NA	Gini_norm	0.068	0.036	1.874	861.570	0.061		
		fixed	NA	MaxHH_norm	−0.051	0.031	−1.634	86.169	0.103		
		ran_pars	Bigregion	sd__(Intercept)	0.561	*NA*	*NA*	NA	*NA*		
		ran_pars	Residual	sd__Observation	1.013	*NA*	*NA*	NA	*NA*		
2	Asia							156.000	1.02E-05		0.144
		fixed	NA	(Intercept)	0.954	0.146	6.548	NA	8.01E-10		
		fixed	NA	DeltaCult_norm	−0.449	0.084	−5.364	NA	2.89E-07		
		fixed	NA	SA_norm	0.515	0.205	2.510	NA	0.013		
		fixed	NA	Gini_norm	0.217	0.149	1.456	NA	0.148		
		fixed	NA	MaxHH_norm	−0.233	0.967	−2.414	NA	0.017		
3	Europe							33.000	0.119		0.085
		fixed	NA	(Intercept)	−0.247	0.122	−2.016	NA	0.052		
		fixed	NA	DeltaCult_norm	0.039	0.107	0.036	NA	0.719		
		fixed	NA	SA_norm	−0.075	0.116	−0.651	NA	0.520		
		fixed	NA	Gini_norm	0.115	0.142	0.805	NA	0.426		
		fixed	NA	MaxHH_norm	0.264	0.149	1.773	NA	0.085		
4	Mesoamerica							310.000	<2.2e-16		0.629
		fixed	NA	(Intercept)	0.073	0.032	2.294	NA	0.022		
		fixed	NA	DeltaCult_norm	−1.135	0.052	−21.937	NA	<2e-16		
		fixed	NA	SA_norm	0.102	0.036	2.807	NA	0.005		
		fixed	NA	Gini_norm	0.017	0.012	1.437	NA	0.152		
		fixed	NA	MaxHH_norm	−0.004	0.010	−0.397	NA	0.691		
5	North America							289.000	<2.2e-16		0.913
		fixed	NA	(Intercept)	−1.858	0.192	−9.670	NA	<2e-16		
		fixed	NA	DeltaCult_norm	−1.687	0.032	−52.597	NA	<2e-16		
		fixed	NA	SA_norm	0.039	0.077	0.506	NA	0.614		
		fixed	NA	Gini_norm	0.061	0.052	1.117	NA	0.240		
		fixed	NA	MaxHH_norm	−2.222	1.057	−2.103	NA	0.036		
6	South America							41.000	1.43E-06		0.496
		fixed	NA	(Intercept)	0.414	0.137	3.015	NA	0.004		
		fixed	NA	DeltaCult_norm	−0.875	0.157	−5.557	NA	1.85E-06		
		fixed	NA	SA_norm	0.034	0.074	0.458	NA	0.650		
		fixed	NA	Gini_norm	−0.071	0.081	−0.874	NA	0.387		
		fixed	NA	MaxHH_norm	0.179	0.087	2.043	NA	0.048		

^*^For models with random effects this is the marginal pseudo-R2, the variance explained by the fixed effects alone.

^†^For models with random effects this is the conditional pseudo-R2, the variance explained by fixed and random effects together. For models with only fixed effects, this is the adjusted R2.

Our approach assumes that residential disparities visible in a particular phase are representative of the disparities across multiple phases of occupation. We refer to this as the “snapshot problem,” in that we have a synchronic “snapshot” of a variable from a particular moment in time which we are comparing with a diachronic variable, duration of occupation, over which it is possible for the snapshot value to change. One way to examine the degree to which the snapshot problem is having an effect would be to examine multiple phases at the same sites to identify the magnitude of changes in residential disparities over time. Unfortunately, there are only 23 sites with multiple phases in our dataset. Several of these are also highly unusual and are unlikely to be representative of general trends. For example, Aşıklı Höyük in western Cappadocia, Türkiye has several phases of occupation during the earliest phases of the Neolithic, but the residential pattern in each phase is identical as houses are rebuilt in precisely the same configuration. Nine sites in Britain were occupied across several phases of the Roman period, or from the Roman to Anglo-Saxon period, but the degree of social upheaval and reorganization occurring at both a site level and across the social and economic sphere at that time is similarly unlikely to be representative of wider trends in our global dataset. As an alternative, we examined the relationship between residence disparities and duration before and after the phase from which our residence dataset was collected (*SI Appendix*, Fig. S5). These are calculated using the start date of the settlement occupation, the mean data of the phase occupation, and the end date of the settlement occupation. The results show that longer durations prior to the snapshot phase are only slightly correlated with lower Gini coefficients on residence disparities, but the regression line is close to flat. This suggests that residential disparities do not rise over time at individual sites. Duration after occupation exhibits a similar very slight negative trend, suggesting neither higher nor lower Gini values are strongly correlated with increased chance of settlement abandonment.

## Discussion

The dynamics which govern individual settlement trajectories are complex and influenced by a wide range of factors, including forms of individual and community-level agency which approximate stochastic processes (37). As the papers in this Special Feature demonstrate, archaeological samples of residential disparities across sites, which we argue are a conservative measure of past material inequality, are subject to similar complexities, and both persistence and residence datasets are impacted by sampling issues and recovery biases resulting from regional archaeological traditions and capacities. Despite all of this, comparing settlement persistence and residential disparities through the Gini coefficient at the scale available through the GINI project dataset does demonstrate a weak positive correlation; more persistent sites tend to be more unequal, and the strength of this relationship grows as societies become more complex. These simple findings present a challenge for concepts of sustainability which seek to promote continuity and equality.

The causal linkages which produce these relationships are challenging to disentangle. We have shown that there is no relationship between prior length of occupation and the degree of material inequality at a site level, which rules out the possibility that simply by persisting settlements become more unequal. Similarly, there is no relationship between material inequality and the timing of subsequent settlement abandonment, meaning it is unlikely that the causal relationship is that material inequality itself facilitates persistence. Instead, we would argue that both inequality and persistence share underlying causal drivers, including functional diversity at a site level, and system effects across sites within a network. Another way of articulating our results is that sites with less homogenous residential size distributions tend to last longer. All other things being equal, we would expect increasing diversity within an economic system to correlate with specialization and agglomeration effects [in short, Smithian growth (38, 39)], leading to both higher overall wealth and more uneven distributions of that wealth due to the differential claims of different members of the economic system. Increased diversity has also been related to stability and resilience in a variety of ecological and human complex systems. A diverse community is more likely to contain functional groups capable of responding to a destabilizing event (40), and hierarchy and specialization can facilitate problem-solving (41, 42). However, for human systems, the relationship between diversity and stability is not straightforward, because the productive organization of heterogenous agents comes at a metabolic cost to the system which increases as a proportion of total energy capture as complexity increases (43). Although beyond the scope of this contribution, we note that this suggests there should be a local optimum level of complexity, and perhaps material inequality, for facilitating persistence, dependent on productive capacities and hierarchies.

While intrasite diversity may account for some of the correlation between persistence and material inequality, comparisons with other variables in the GINI project database demonstrate relations *between* sites are also important. Our results show that larger settlements in larger systems tend to be both more persistent and more unequal and importantly that as systems increase in size ([NOfLevels] increases), the strength of this relationship increases (Fig. 2). This suggests that urbanization and the capacity to extract agricultural surplus and other forms of wealth from smaller rural sites enhanced persistence and increased material inequality at larger sites. A greater per capita claim on goods produced by the wider system would be beneficial for persistence, since in times of need larger centers would be able to prioritize their own welfare while, all other things being equal, larger surpluses should also translate into greater distributional differences, and therefore inequality (44). The form this extraction might take varies across the societies and settlements included in our dataset but can be broadly categorized in political and economic terms (45, 46). Larger centers are more likely to be home to political power, and therefore institutions capable of extracting wealth from a hinterland through levies such as taxation, tribute, or tariffs (47). They are also more likely to be economic centers, providing both services which are not available in hinterland settlements (48) and larger aggregate demand compared to hinterland settlements with smaller populations. Identifying which of these mechanisms is in play through the archaeological record is often difficult, but we can see evidence of differential claims on resources in favor of the inhabitants at larger sites from the very earliest phases of “urban” development in regions such as Southwest Asia (49, 50). By the Roman period, concentrations of military and state power may have facilitated the emergence of genuine consumer cities [sensu (51)], with elites capable of extracting surpluses from rural hinterlands on highly unequal terms (52).

Comparison between the different macroregions demonstrates the increasing importance of these settlement system effects as societies grow. In North America, we have fewer large systems and large sites, while in Asia much of our dataset comes from societies with well-established hierarchies. The SA score in North America is strongly negatively correlated with persistence, and the model results have the highest explanatory power. This may be because the absence of system effects means that sites are behaving in the same ways in relation to the chosen variables, while in Asia shifts in some variables, such as site size (captured in [MaxHH]), would have different effects on the persistence of a specific site depending on its position in the hierarchy, reducing the overall model fit. In Mesoamerica and South America, which also include large systems, smaller sites in the hierarchy are not well represented in our sample due to recovery biases, resulting in an imperfect reflection of the total system but a better model fit.

## Outlook

We have shown that as past societies grow more complex and start to look more like our own highly urbanized and interconnected world, the two sides of sustainability, persistence, and equality, can come into conflict. We argue that increasing extractive capacity for larger centers enhances survival prospects and raises material inequality at the same time. One thing we have not been able to test is whether this enhanced capacity comes at the expense of smaller sites, such that their persistence decreases, or whether it is instead a benefit of agglomeration and network effects within larger systems. Assessing this would require persistence data for full settlement systems, which are available in many regions of the world but are not collated in the GINI dataset. The answer to this question has profound implications, since it would allow us to understand whether urbanization enhances sustainability overall or simply redistributes it unevenly across a settlement system. Put another way, for societies at particular scales, a modest increase in material inequality may be a price worth paying if it raises persistence across a settlement system. The next question would then be how much inequality is appropriate for maximum persistence at a given scale? Given the wide variety of both structural and contingent factors which can affect individual settlement trajectories, we would require a dataset far larger than even the GINI project database to identify such an optimum, but it is a theoretical possibility. We would stress here that our argument should not be taken to mean that interventions to reduce material inequality in the present day are doomed to reduce systemic longevity. Indeed, one implication of the lack of a direct causal relationship between persistence and material inequality in our results is that measures can be designed to affect one and not the other. However, we have demonstrated that connections between complexity, inequality and durability exist, and these may provide constraints on possible futures which at the least need to be better understood and potentially negotiated. It may be, and perhaps is most likely the case, that current levels of inequality exceed those required to enhance persistence for societies of the scale and complexity found today. We would also note that our database includes outliers from the general trend, particularly large, long-lived sites with low Gini coefficients, such as Monte Albán, Teotihuacan, Athens, and Mohenjo-daro, which could provide more optimistic models. These well-studied cases from diverse eras in the past are noted as exceptional across many of the variables in the analyses conducted across this Special Feature (for example, refs. 46 and 53) and here illustrate that the seeming link between community persistence and material inequality could be institutionally muted.

In common with several of the other papers in this Special Feature (54–56), our work demonstrates that the spatial scale of analysis at which inequality is analyzed is extremely important. Here, we have also shown this to be the case for persistence. In very broad terms, we might say that as systems increase in scale, the degree of information about the system which can be inferred from a single site decreases. The implication of this is that we should assess both persistence and inequality across multiple social and spatial scales appropriate to the forms of the systems we are interested in refs. 57 and 58. This plays to one of the strengths of the residential disparities approach, in that it can be scaled up very easily by aggregating residences from multiple sites. Here, the limitation is the quality of the dataset available through the archaeological record. In contrast, identifying the persistence of systems beyond sites, such as states or empires, means grappling with definitional challenges around the form and magnitude of social change required to constitute the start or end of a particular formation (59). If you ask four Classicists when the Roman Empire ended you are likely to get five different answers. The nature of political relations also varies across space, often within single polities in preindustrial societies (60). Economic and social systems are, if anything, even harder to pin down, although one approach might be to focus on the tempos of change across the sorts of interaction spheres laid out by Green et al. in this Special Feature (56).

## Materials and Methods

### GINI Project Data.

All of the archaeological data on residential unit area, site area, site size, number of households, number of levels, and position within levels derive from the GINI database (25). The database was constructed by a team of regional experts and was designed to capture the full range of domestic features and site types known from the archaeological record at a global scale. A range of factors, including past social practices, environmental constraints, preservation conditions, and archaeological research trajectories, introduce sampling biases (27). These are not uniform or systematic across the dataset and often vary by region. For example, in the US Southwest, Europe, and Japan, larger sites are likely underrepresented, while in Mesoamerica, they may be overrepresented. In Southwest Asia, large sites have received more attention, but excavations have focused on major public buildings and urban infrastructure. As a result, much of our evidence for domestic architecture comes from smaller sites. We have sought to mitigate these biases through a broad sampling strategy and appropriate statistical rigor. Several workshop meetings were held across the project to ensure standardization in coding variables and definitions, including fundamental questions such as how to identify and delineate residences and sites cross-culturally. Further details on these decisions are available in other papers in this Special Feature [particularly (27, 29, 54)]. Data on persistence were not available in the GINI database for sites from several regions and one macroregion (Africa), and we have excluded these sites from our analysis (*SI Appendix*, Table S1).

### Settlement Persistence.

We recorded settlement persistence by identifying the duration of substantial and permanent occupation of a site before, during, and after the phase from which our residence data was recorded. This means, for example, that if a site was occupied, then abandoned, and then reoccupied, and we recorded residence sizes during the reoccupation, duration would only be recorded for the second occupation. If a site changes significantly in size or importance but remains substantially occupied, the duration would continue until total abandonment. However, we excluded excavation phases which only revealed ephemeral traces of settlement relative to other phases (sometimes termed “squatter occupation”). This approach does mean that we may miss contractions or expansions in site size which could be relevant for understanding past social trajectories. This sort of data is not widely available for sites across our dataset and would best be investigated by regional studies dealing with fewer settlements.

Identifying substantial and permanent occupation relies on knowledge of local archaeological sequences, methods, and context and as such was undertaken by the regional experts. For the majority of the dataset, excavation and stratigraphy were used to identify the nature of settlement but in some regions, particularly in Mesoamerica, permanence was established using surface pottery.

## Supplementary Material

Appendix 01 (PDF)

## Data Availability

All scripts (in R) and data for replicating the analyses and reproducing main and supplementary figures are stored on the tDAR repository under The Global Dynamics of Inequality (GINI) Project, tDAR id: 496853 (61).
