# Supplementary material for: Housing inequality and settlement persistence are associated across the archaeological record
Source: Proc Natl Acad Sci U S A. 2025 Apr 14;122(16):e2400696122. doi: 10.1073/pnas.2400696122 (PMC12036995; doi:10.1073/pnas.2400696122)
Supplement: Supplementary file 1 — Appendix 01 (PDF) [file pnas.2400696122.sapp.pdf]

PNAS

Supporting Information for

Housing inequality and settlement persistence are associated across the archaeological record

Dan Lawrence[1*], Amy Bogaard[2, 3], Gabriela Cervantes Quequezana[4], Francesca Chelazzi[5], Gary M. Feinman[6, 7], Adam S. Green[8], Helena Hamerow[2], Jessica Munson[9], Scott G. Ortman[3], Amy E. Thompson[10]

1 Department of Archaeology, Durham University, South Road, Durham, DH1 4HW

2 School of Archaeology, University of Oxford, Oxford OX1 3TG, UK

3 Santa Fe Institute, 1399 Hyde Park Rd., Santa Fe, NM 87501

4 Department of Anthropology, University of Pittsburgh, PA 15260 USA

5 Department of Asian and North African Studies, Ca' Foscari University, Venice, 30123, Italy

6 Neguanee Integrative Research Center, Field Museum of Natural History, Chicago, IL 60605, USA

7 Department of Anthropology, University of Illinois-Chicago, Chicago, IL 60607, USA

8 Department of Archaeology and Department of Environment and Geography University of York, King's Manor, York, YO1 7EP, United Kingdom

9 Department of Anthropology-Sociology, Lycoming College, Williamsport, PA 17701, USA

10 Department of Geography and the Environment, The University of Texas at Austin, Austin, TX, 78712, USA

*Dan Lawrence

Email: dan.lawrence@durham.ac.uk

This PDF file includes:
Supporting text
Figures S1 to S5
Table S1

Additional Graphs and Table

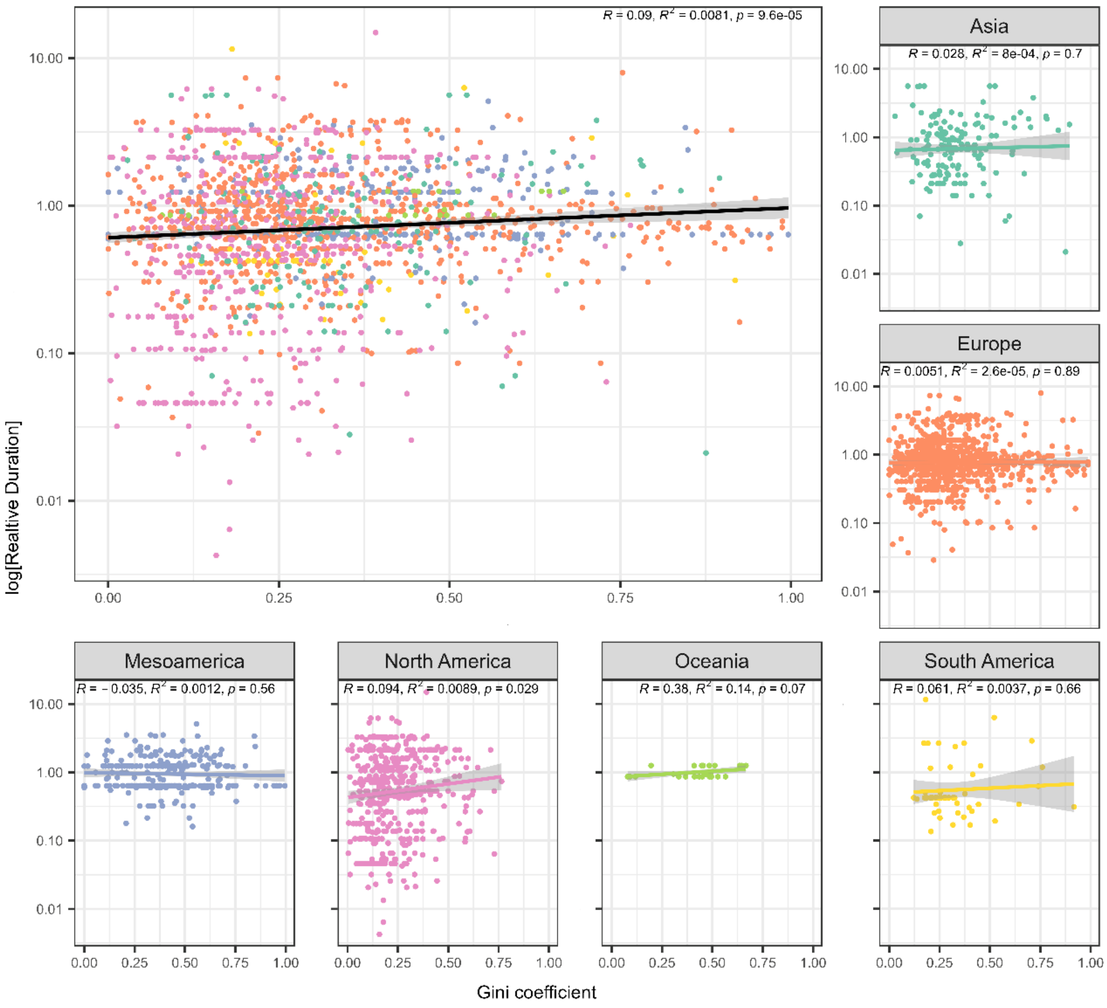

Fig. S1. Scatter plots of Gini values and relative durations of occupation (persistence) overall and by macroregion

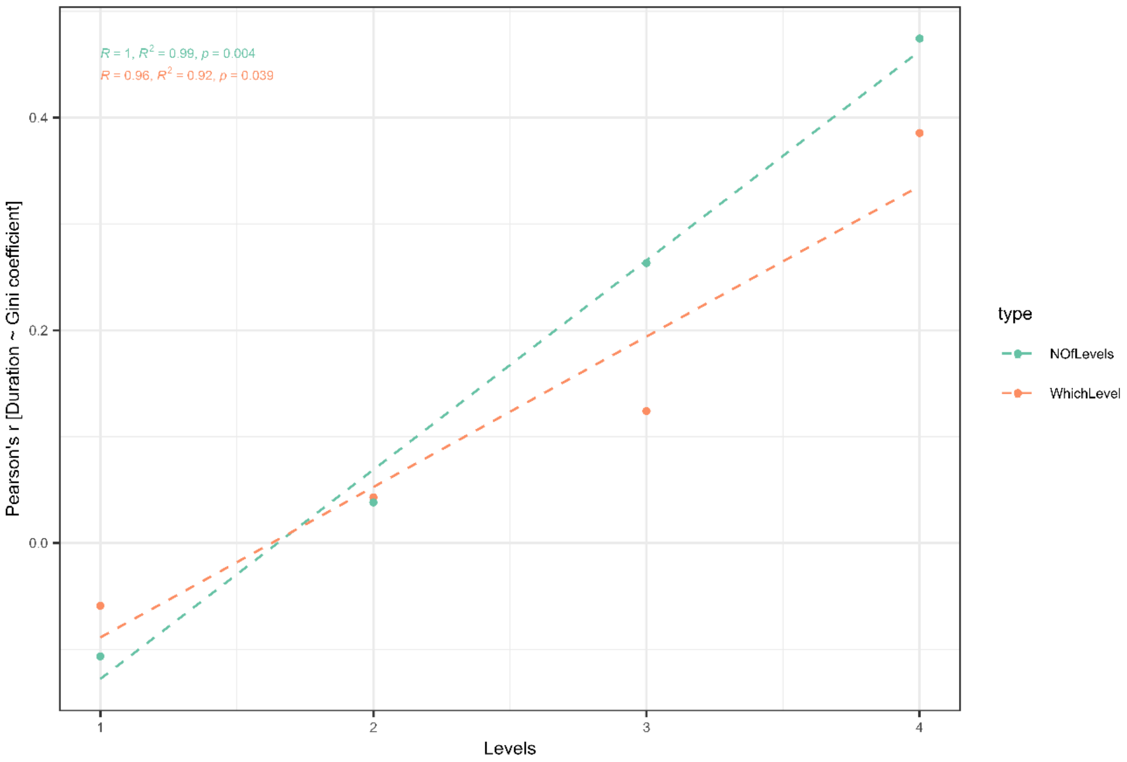

Fig. S2. Correlation Coefficients for slopes of relative durations of occupation (persistence) by [WhichLevel] and [NOfLevels]. Note Levels 5 and 6 groups not shown (see Figures 2 and 3 in main manuscript for explanation)

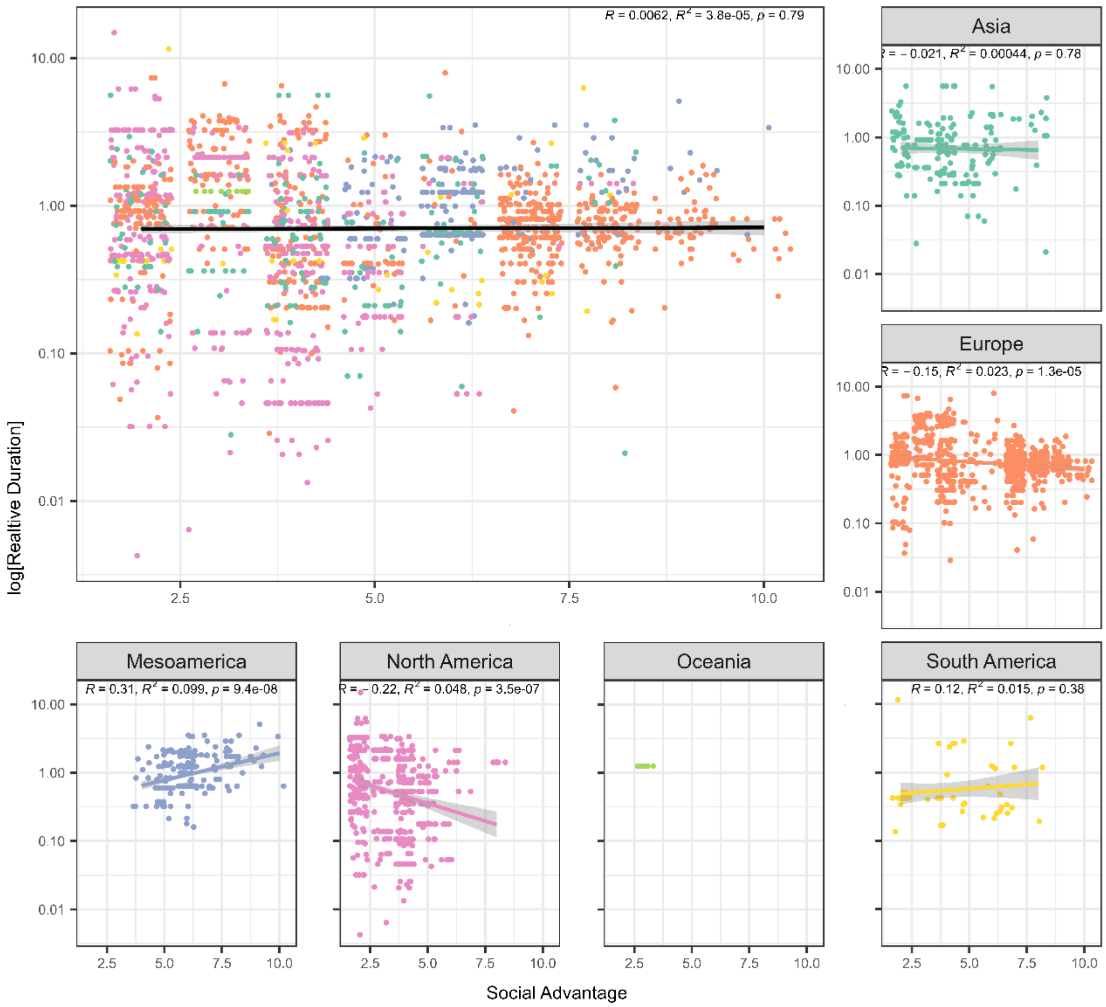

Fig. S3. Scatter plots of the log of relative duration of occupation (persistence) and Social Advantage (SA) metric ([NOfLevels] + [WhichLevel]) overall and by Big Region

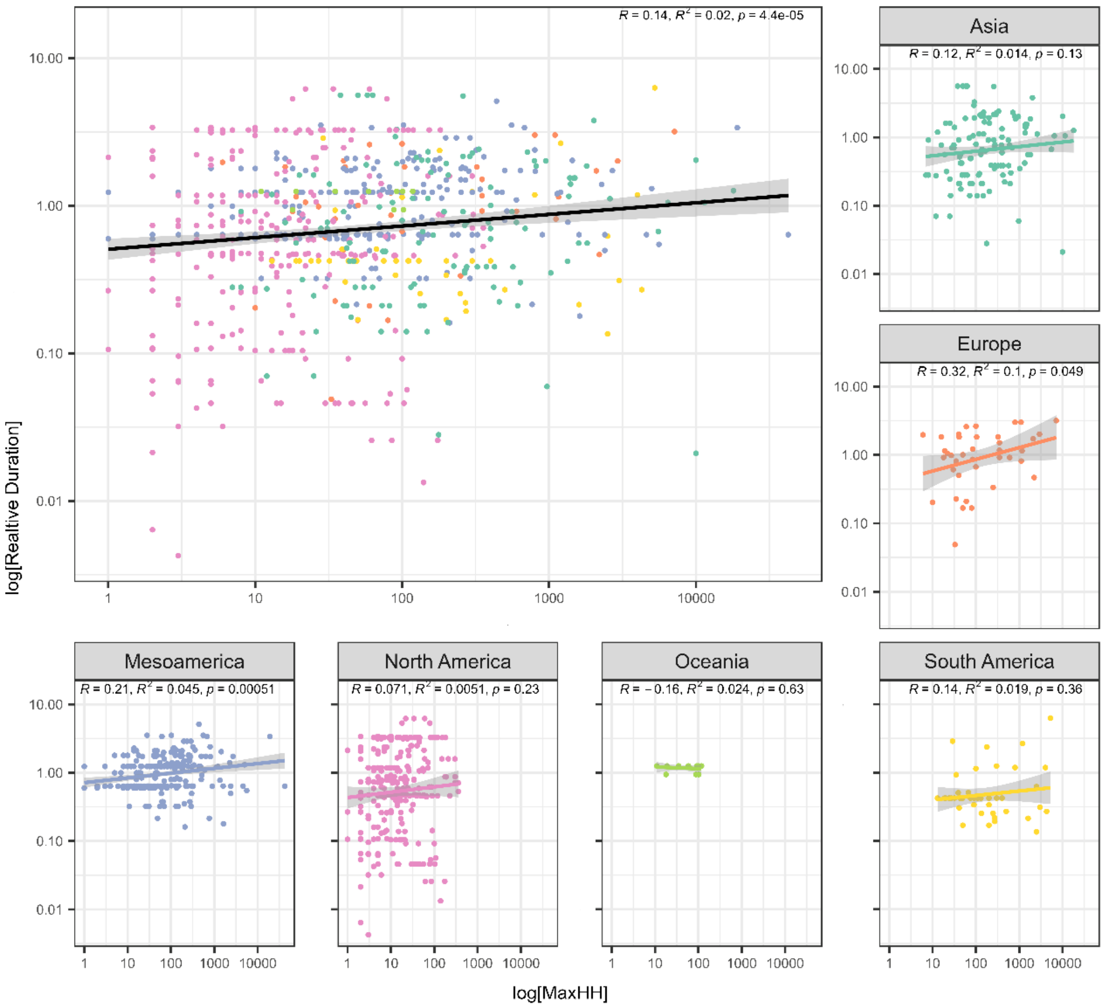

Fig. S4. Scatter plots of the log of relative duration of occupation (persistence) and maximum number of households per site [MaxHH] overall and by Big Region

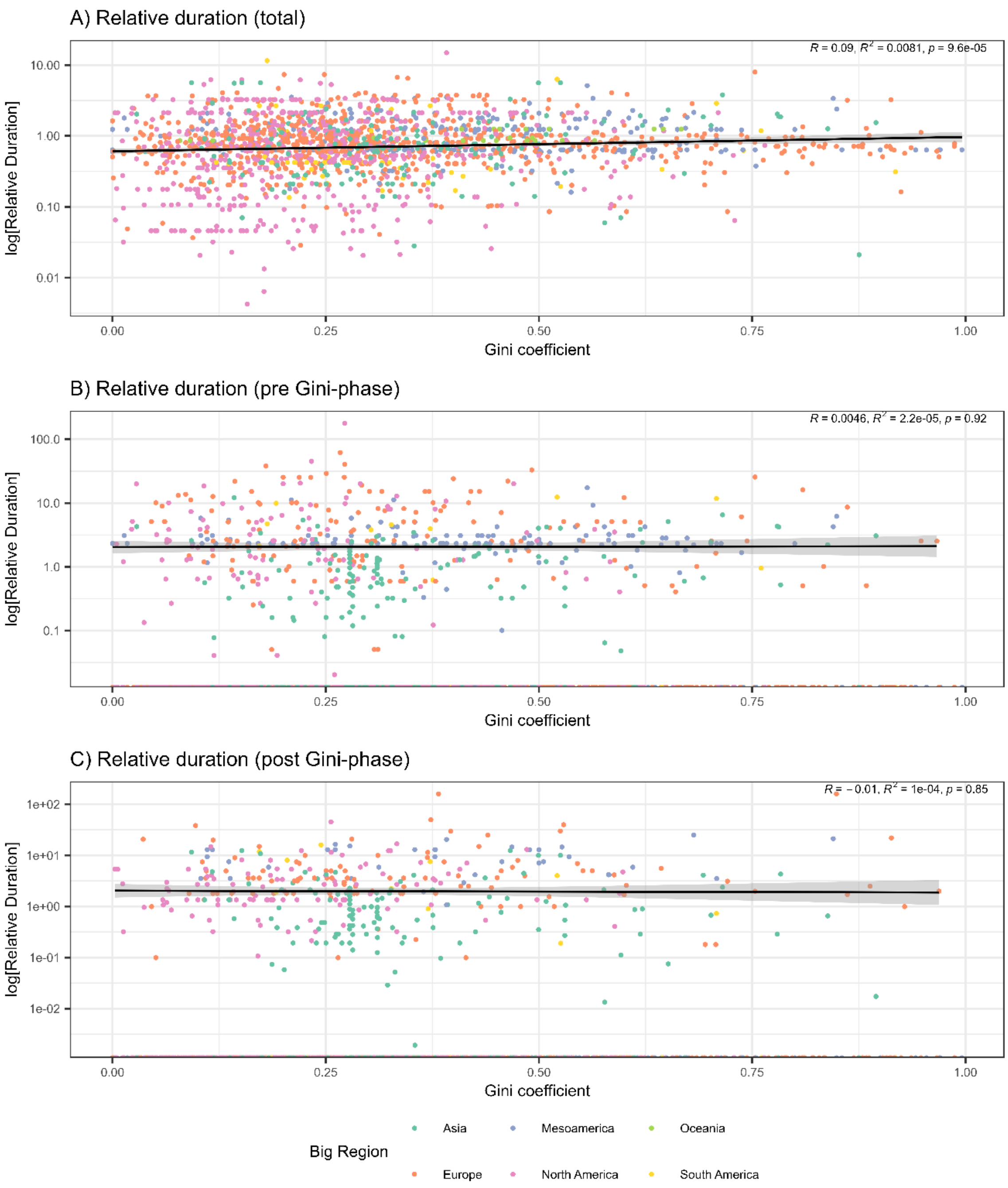

Fig. S5. Scatter plots exploring the relationship between the log of relative duration of occupation and the Gini coefficient. A) uses the total relative duration, B) uses the relative duration before the midpoint of the phase in which the residential data was collected, C) uses the relative duration after the midpoint of the phase in which the residential data was collected

Macroregion	Region	Count of sites	Count of Site Phases	Duration mean (region)	Duration mean (macro region)	MaxHH mean (region)	MaxHH mean (macro region)
**Africa**	*E Africa*	15	11	NA	426	54	50
	Horn of Africa	9	2	NA		33	
	N Africa	1	1	426		NA	
	S Africa	95	21	NA		54	
	W Africa	50	13	NA		NA	
**Asia**	*E Asia*	344	5	668	1273	92	1176
	S Asia	31	13	895		3668	
	W Asia and Cyprus	145	22	1425		685	
**Europe**	*C Europe*	38	7	63	477	160	511
	E Europe	38	6	174		1259	
	Great Britain	1115	13	491		386	
	SE Europe	124	14	597		623	
	W Europe	15	3	14		18	
**Mesoamerica**	*Central Mexico*	564	10	502	541	213	237
	Maya	52	7	932		339	
	Southern Mexico	9	2	1033		1144	
**North America**	*Great Plains*	178	13	2177	734	42	23
	Northeast NA	45	3	42		14	
	Northwest NA	99	14	766		8	
	Southeast NA	153	11	282		9	
	Southwest NA	606	21	469		82	
**Oceania**	*Melanesia*	26	1	NA	638	25	36
	Polynesia	27	1	638		58	
**South America**	*Central Andes*	40	10	481	562	2215	721
	Southern Andes	41	7	590		222	

Table S1. Settlement Counts, Phases and Mean Durations by macroregion and region
